# Different Types of Coagulase Are Associated With 28-Day Mortality in Patients With *Staphylococcus aureus* Bloodstream Infections

**DOI:** 10.3389/fcimb.2020.00236

**Published:** 2020-05-19

**Authors:** Matthias Karer, Manuel Kussmann, Franz Ratzinger, Markus Obermueller, Veronika Reischer, Heidemarie Winkler, Richard Kriz, Heinz Burgmann, Bernd Jilma, Heimo Lagler

**Affiliations:** ^1^Division of Infectious Diseases and Tropical Medicine, Department of Medicine I, Medical University of Vienna, Vienna, Austria; ^2^Department of Clinical Pharmacology, Medical University of Vienna, Vienna, Austria; ^3^Division of Medical and Chemical Laboratory Diagnostics, Department of Laboratory Medicine, Medical University of Vienna, Vienna, Austria; ^4^Ihr Labor, Medical Diagnostics Laboratories, Vienna, Austria

**Keywords:** sepsis, bacteremia, coagulation, fibrinogen, cluster analysis, virulence factors, adhesin

## Abstract

**Background:** *Staphylococcus aureus (S. aureus)*, a leading cause of bacteremia and infective endocarditis, exploits the human coagulation system by using a wide range of specific virulence factors. However, the impact of these host-pathogen interactions on the outcome of patients with *Staphylococcus aureus* bacteremia (SAB) remains unclear.

**Methods:** A total of 178 patients with *S. aureus* bacteremia were included and analyzed regarding bacterial factors (*coa* gene size, *vWbp, clfA, clfB, fnbA, fnbB, fib)* and clinical parameters. A stepwise multivariate Cox regression model and a Partitioning Around Medoids (PAM) cluster algorithm were used for statistical analysis.

**Results:** Patients' risk factors for 28-day mortality were creatinine (OR 1.49, *p* < 0.001), age (OR 1.9, *p* < 0.002), fibrinogen (OR 0.44, *p* < 0.004), albumin (OR 0.63, *p* < 0.02), hemoglobin (OR 0.59, *p* < 0.03), and CRP (OR 1.72, *p* < 0.04). Five distinct bacterial clusters with different mortality rates were unveiled, whereof two showed a 2-fold increased mortality and an accumulation of specific coagulase gene sizes, 547-base pairs and 660-base pairs.

**Conclusions:** Based on the data obtained in the present study an association of coagulase gene size and *fib* regarding 28-day mortality was observed in patients with *S. aureus* bloodstream infections. Further animal and prospective clinical studies are needed to confirm our preliminary findings.

## Introduction

*Staphylococcus aureus* (*S. aureus*) is one of the main bacterial pathogens in humans causing potentially severe infections with a high mortality such as bacteremia, infective endocarditis as well as pleuropulmonary- and device-related infections (Diekema et al., 2001). Especially age at either extreme of life, renal insufficiency, chronic liver disease, immunosuppression, Human Immunodeficiency Virus infection, hemodialysis, unknown primary focus and a history of cancer are risk factors for an increased mortality in *S. aureus* bloodstream infections (Tong et al., 2015; Braquet et al., 2016). In the last decades extensive work has been undertaken to discover bacterial genetic factors as independent predictors of mortality in *S. aureus* bacteremia (SAB) which often resulted in diverging results (Fowler et al., 2005; Miller et al., 2012; Gasch et al., 2014).

Some of these bacterial genetic factors which interact with the coagulation system, namely coagulase (*coa*), von Willebrand factor-binding protein (*vWbp*), clumping factor A and B (*clfA, clfB*), fibronectin-binding protein A and B (*fnbA, fnbB*) and fibrinogen binding protein (*fib*), have mainly been studied *in vitro* and in animal models (Kerdudou et al., 2006; Vanassche et al., 2013; Cardile et al., 2014; McDonnell et al., 2016; Wang et al., 2017; Mancini et al., 2018). Strains with the *coa* and *clfA* genes have shown a considerable impact on infection onset and progression in mouse and rat models of catheter infections and infective endocarditis, respectively (Vanassche et al., 2013; McDonnell et al., 2016; Wang et al., 2017; Mancini et al., 2018).

Furthermore, Coa acts in synergy with the newly discovered van Willebrand factor-binding protein, by conversion of fibrinogen to fibrin, which may result in severe infective endocarditis (McAdow et al., 2012; Claes et al., 2017). In addition, recent work showed that the tandem repeat region of the *coa* gene represents the motive necessary for binding of fibrinogen and, therefore, may participate in the turnover of fibrinogen to fibrin (Ko et al., 2016).

Studies investigating a potential impact of different virulence factors on mortality in SAB did not discriminate different isoforms of coagulase and mainly used the *coa* gene for assessment of bacterial relatedness rather than a clinical outcome parameter (Fowler et al., 2005; González-Domínguez et al., 2014).

Thus, the present study sets out to investigate risk factors for 28-day mortality and further implemented genetic factors of *S. aureus* isolates to discover distinct genetic combinations influencing mortality in SAB.

## Materials and Methods

### Patients' Characteristics

For this monocentric retrospective cohort study, a total of 683 episodes of SAB, occurring from 2012 to 2015 at the Vienna General Hospital, Austria, a tertiary care hospital, were screened from an available in-house database. A total of 178 SAB episodes could be included in an age-stratified random sample. The medical history of all patients was assessed until 1-year after dismissal or patient's death. Laboratory data, vital parameters, medication, and co-morbidities were obtained at the time of blood culture acquisition, which was also the start of the observation period. The primary endpoint of this study was the influence of bacterial virulence factors on the 28-day all-cause mortality. Co-morbidities and received medications were defined as stated in the Supplementary Material.

*S. aureus* isolates were gathered from routinely performed blood cultures and stored at −80°C until analysis. Infections were classified according to definitions of the European Centre for Disease Prevention and Control (ECDC) point prevalence survey of healthcare-associated infections (European Centre for Disease Prevention Control, 2015). Time to positivity was defined as the time from the beginning of blood culture incubation to the detection of bacterial growth. If more than one blood culture bottle became positive, the shortest time was used. Adequate antimicrobial therapy was defined as the initiation of at least one antimicrobial substance to which the isolated strain showed *in vitro* susceptibility according to the clinical breakpoints of the European Committee on Antimicrobial Susceptibility Testing (EUCAST) (The European Committee on Antimicrobial Susceptibility Testing. Breakpoint tables for interpretation of MICs and zone diameters. Version 9.0, 2019).

### Genetic Analysis

A modified coagulase typing was performed based on the methods described by Hookey et al. (1998). Briefly, acquired isolates were incubated overnight on 5% sheep blood agar plates (bioMérieux, France) at 37°C and bacterial colonies were suspended in 200 μL water. Samples were heated up to 95°C for 10 min and sonicated for 15 min. A PCR for the *coa*-gene was conducted using Taq-polymerase (Applied Biological Materials Inc., Canada). Samples were analyzed immediately using gel electrophoresis and restriction fragment length polymorphism (RFLP) analysis using AluI (Roche Diagnostics, Germany). Clotting factors were determined for each isolate using a multiplex PCR, and the *vWbp* gene was determined using a singleplex PCR as described by Ghasemian et al. (2015) and Sukhumungoon et al. (2014), respectively (Sukhumungoon et al., 2014; Ghasemian et al., 2015). Primers, PCR and RFLP conditions used in the present study are described in the Supplementary Material (see Table S1 and Supplementary Data). For fragment detection, a 2% agarose gel with PepGreen (Peqlab, Germany) was used. Gels were assessed by three independent investigators and experiments were repeated if one of the three investigators observed another result. The standardized *S. aureus* ATCC33592 served as a positive control.

### *In vitro* Biofilm Formation

*In vitro* biofilm formation capacity was determined for each isolate using a crystal violet assay. Briefly, all bacterial strains were incubated overnight on 5% sheep blood agar plates (bioMérieux, France) at 37°C and suspended in tryptic soy broth (TSB; Oxoid, ThermoFisher Scientific) to a concentration equivalent to a 0.5 McFarland standard. Ninety-six well polystyrene flat-bottomed microtiter plates (Cellstar®, Greiner bio-one®, Frickenhausen, Germany) were filled with 200 μL each and incubated at 37°C for 24 h. Planktonic cells were removed using phosphate-buffered saline (PBS), and biofilm was heat-fixed for 10 min at 60°C and subsequently fixed with 150 μl methanol (Merck, Darmstadt, Germany). Plates were dried at room temperature, stained for 20 min using 150 μl 1% crystal violet (Merck KGaA®, Darmstadt, Germany), and washed with tap water. For improved quantification, 150 μl 33% acetic acid (AnalaR Normapur, Prolabo®, VWR International®, USA) was placed in each well, and plates were incubated at 37°C and 50% humidity for 1 h. Plates were measured using a microplate reader (Sunrise, Tecan, Switzerland) at 595 nm with a reference measurement at 405 nm. The mean OD for each isolate was determined by measuring 14 replicates in two separate plates (7 replicates each).

### Statistical Analysis

For the statistical analysis, R Version 3.6.1 (Vienna, Austria) was applied. Numeric data are given as median with 1st and 3rd quartiles. Categorical data are presented as count with their percentage. A stepwise multivariate Cox regression model including age, sex, Body Mass Index (BMI), intensive care unit (ICU) admission, diabetes, plasma coagulation inhibiting medication and laboratory parameters including C-reactive protein (CRP), hemoglobin, fibrinogen, creatinine, albumin, gamma-glutamyl transferase, bilirubin, platelet count and white blood cells was calculated in order to assess the effect of demographic and laboratory markers at the time point of study inclusion on the 28-day survival rate (Chambers and Hastie, 1992). Using both, a forward and backward search strategy, the variable set minimizing the Akaike information criterion (AIC) was chosen. Moreover, Kaplan-Meier plots were used to display the relationship between patient age, laboratory parameters, and the survival rate. Age and creatinine were dichotomized according to an optimal cut-off point for identifying high-risk patients by using the maximally selected rank statistics measure according to Hothorn and Lauser (2002) (implemented maxstat R package) (Hothorn and Lauser, 2002). To find meaningful bacterial genetic clusters among the bacterial isolates, the PAM (Partitioning Around Medoids, R package: cluster Version 2.1) cluster algorithm was applied using the following variables: *in vitro* biofilm formation capacity, MRSA, *fnbB, fnbA, fib, vWbp*, coagulase gene size, *clfA, clfB*, coagulase RFLP Type. Prior to analysis, the Gower distance measure, to evaluating dissimilarities between isolates (implemented in the daisy function, cluster R package) was used for assessing the optimal number of clusters (*k* = 2 to 20), which were found for *k* = 5 (Reynolds et al., 1992). To test significant differences of baseline characteristics between clusters, the Kruskal-Wallis test and Fisher's exact test were used. *P*-values lower than 0.05 were considered to be statistically significant. The Bonferroni-Holm correction was applied to correct for an accumulation of an error related to multiple testing. High and low risk mortality groups were formed from the clusters and evaluated by means of a Fisher's Exact test (with a one-sided significance level, as it is a directional hypothesis).

## Results

### Patients' Characteristics

A total of 178 patients were included in this analysis. The median age was 59 years (Q1–Q3: 43–71) with 121 (68%) male patients, an intensive care unit (ICU) admission rate of 27% (*N* = 46) and a methicillin-resistant *Staphylococcus aureus* (MRSA) rate of 7% (*N* = 13). The overall accurate antimicrobial prescription rate was 92% (*N* = 163) at time of index blood culture acquisition. According to ECDC criteria, most infection foci remained unclear (48%, *N* = 86) followed by catheter-related (17%, *N* = 31) and pleuropulmonary infections (10%, *N* = 18). The most common co-morbidities were arterial hypertension (57%, *N* = 102), heart disease (42%, *N* = 75), and renal impairment (33%, *N* = 59). In this cohort, one out of four patients had cancer with 19% (*N* = 34) solid cancer patients and 6% (*N* = 10) patients with different types of hematologic diseases (see Table 1).

**Table 1:** Patients' characteristics.

**Baseline characteristics (*****n*** ***=*** **178)**^**A**^			
Age—median years (Q1–Q3)	59 (43–71)	Anticoagulation—*N* (%)	46 (26%)
Male gender—*N* (%)	121 (68%)	Platelet inhibitors—*N* (%)	57 (32%)
BMI—median (Q1–Q3)	25 (22–25)	**Co-morbidities—*****N*** **(%)**	
ICU admission—*N* (%)	46 (26%)	Hypertension	102 (57%)
28 day mortality—*N* (%)	29 (16%)	Heart disease	75 (42%)
Adequate antimicrobial treatment^B^–*N* (%)	163 (92%)	Renal impairment	59 (33%)
Time to positivity—median hours (Q1–Q3)	11 (6–16)	Diabetes	41 (23%)
MRSA—*N* (%)	13 (7%)	Malignancy	34 (19%)
**Genetic markers—*****N*** **(%)**		Hypothyroidism	28 (16%)
*fnbA*	148 (83%)	Liver cirrhosis	28 (16%)
*fnbB*	40 (22%)	COPD	17 (10%)
*clfA*	174 (98%)	Autoimmune disease	16 (9%)
*clfB*	173 (97%)	i.v. drug abuse	14 (8%)
*fib*	84 (47%)	Leukemia	10 (6%)
*Vwbp*	170 (96%)	Solid-organ transplant	10 (6%)
**Infection focus (ECDC)**^**C**^**–*****N*** **(%)**		**Laboratory parameters -median (Q1–Q3)**	
Unknown source	86 (48%)	CRP (mg/dL)	12 (6–23)
Catheter-related	31 (17%)	White blood cell count (G/L)	9.9 (6.6–13.7)
Pulmonary infection	18 (10%)	Platelets (G/L)	159 (106–248)
Surgical site infection	9 (5%)	Hemoglobin (g/dL)	10.4 (9.2–12)
Endocarditis	6 (3%)	Creatinine (mg/dL)	1.1 (0.8–1.9)
Bone infection	5 (3%)	Blood urea nitrogen (mg/dL)	21.3 (14.3–21.2)
Joint infection	5 (3%)	Fibrinogen (mg/dL)	493 (359–633)
Urinary tract infection	5 (3%)	Prothrombin time (%)	72 (49–89)
Digestive tract infection	2 (1%)	Albumin (g/L)	30.5 (26.5–36.8)
		Total Bilirubin (mg/dL)	0.7 (0.5–1.4)

BMI, Body Mass Index, ICU, Intensive care unit; MRSA, Methicillin-resistant Staphylococcus aureus; COPD, chronic obstructive pulmonary disease; coa, coagulase; vWbp, von Willebrand factor-binding protein; clfA and clfB, clumping factor A and B; fnbA and fnbB, fibronectin-binding protein A and B; fib, fibrinogen-binding protein; ECDC, European Center of Disease Control; CRP, C-reactive protein.

A Q1–Q3: Quartile 1 to Quartile 3.

B Adequate antimircobial treatment: defined as the initiation of at least one antimicrobial substance to which the isolated strain showed in vitro susceptibility according to the clinical breakpoints of the European Committee on Antimicrobial Susceptibility Testing (The European Committee on Antimicrobial Susceptibility Testing. Breakpoint tables for interpretation of MICs and zone diameters. Version 9.0, 2019) (EUCAST).

C * European Centre for Disease Prevention and Control (ECDC) point prevalence survey of healthcare-associated infections (European Centre for Disease Prevention Control, 2015)*.

### Survival Analysis

**Figure 1:**
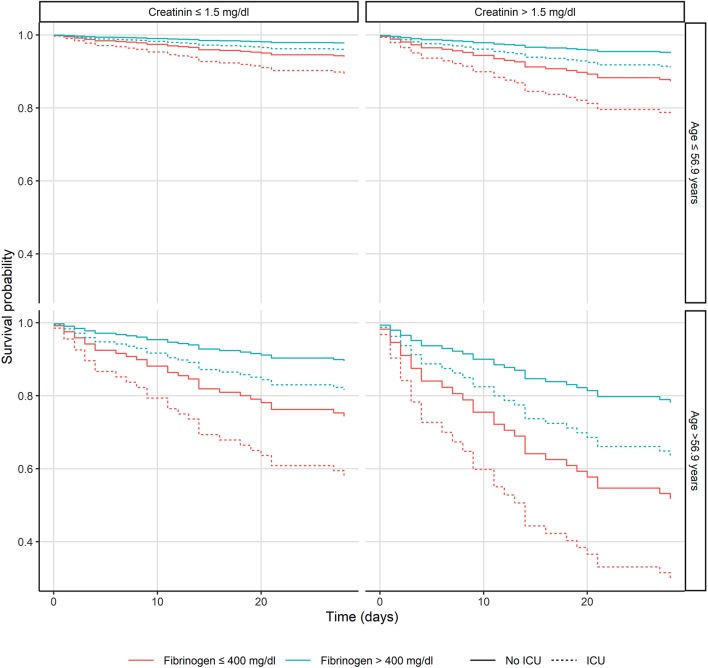
Kaplan-Meier plots of the patients with *Staphylococcus aureus* bacteremia presenting the relationship between age, creatinine and fibrinogen levels and intensive care unit admission. ICU, Intensive care unit.

In a multivariate Cox model, we assessed clinical and laboratory predictors for 28-day mortality at the time of admission. Independent predictors of 28-day mortality were creatinine (OR 1.49, 95% CI: 1.23–1.8, *p* < 0.001), age (OR 1.9, 95% CI: 1.26–2.88, *p* < 0.002), fibrinogen (OR 0.44, 95% CI: 0.25–0.77, *p* < 0.004), albumin (OR 0.63, 95% CI: 0.42–0.94, *p* < 0.02), hemoglobin (OR 0.59, 95% CI: 0.37–0.95, *p* < 0.03), and CRP (OR 1.72, 95% CI: 1.04–2–85, *p* < 0.04). Figure 1 presents the relationship between age (dichotomized, cut-off: 56.9-years), creatinine (dichotomized: 1.5 mg/dl), fibrinogen (dichotomized: 400 mg/dl), and ICU admission on the 28-day mortality (see Figure 1).

### Bacterial Factors

To elucidate the host-pathogen interplay in patients with invasive *S. aureus* infections, different genes and gene variants influencing human coagulation (*clfA, clfB, fnbA, fnbB, fib, vWbp*, and *coa*) as well as methicillin susceptibility and *in vitro* biofilm formation capacity were included as variables within the clinical Cox model. None of these variables demonstrated a significant impact on the 28-day mortality model.

### Cluster Analysis of Bacterial Genetic Factors

**Figure 2:**
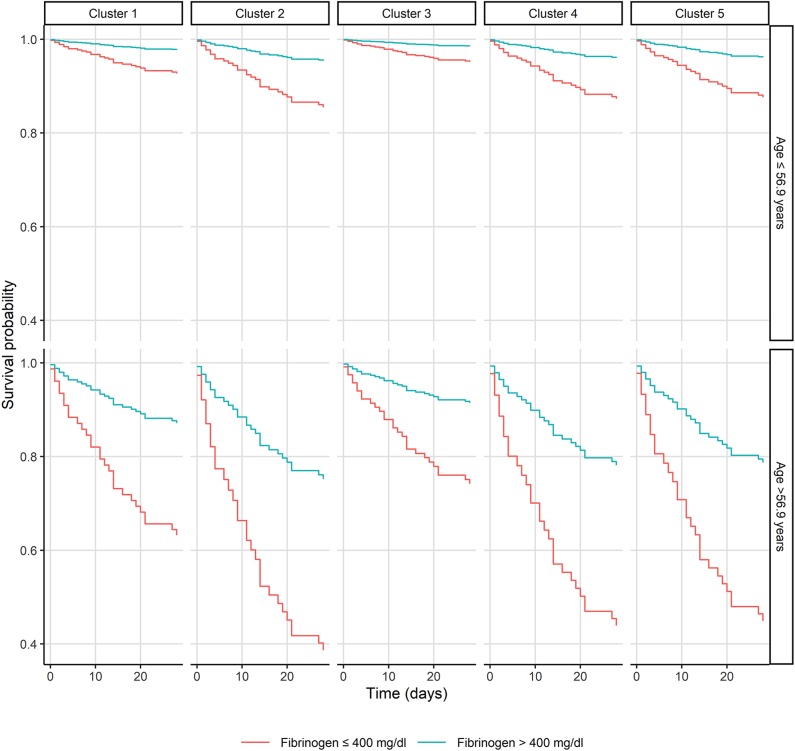
Kaplan-Meier plot of patients with *Staphylococcus aureus* bacteremia in respect of the bacterial genetic clusters, age, and fibrinogen.

A cluster analysis solely using bacterial genetic factors was performed to investigate their influence on 28-day mortality. Five distinct clusters were generated with two clusters displaying a 28-day mortality of 12 and 10% [cluster 1 (*n* = 43) and 3 (*n* = 40), respectively] and three clusters with mortality rates of 20, 21, and 23% [cluster 2 (*n* = 35), 4 (*n* = 29) and 5 (*n* = 31), respectively] (see Figure 2). Microbiological data, including bacterial genetic factors as well as patients' characteristics, are displayed in Table 2 and the Supplementary Material (see Figure S1). ICU admission rates varied between clusters ranging from 17 to 37%. MRSA infection rates were 23% in cluster 2, whereas in other clusters MRSA rates were ≤ 5%. In cluster 1, 2, and 5, the most prominent *coa* gene size was 660 base pairs (bp) with 40, 86, and 77% of isolates, respectively. In cluster 3, the 603 bp and in cluster 4, the 547 bp *coa* gene occurred most frequently, with 78 and 97% of isolates, respectively [see Table 2 and Supplementary Material (Figure S2)].

**Table 2:** Baseline characteristics of calculated clusters from patients with *Staphylococcus aureus* bacteremia.

	**Cluster 1** **(*n =* 43)**	**Cluster 2** **(*n =* 35)**	**Cluster 3** **(*n =* 40)**	**Cluster 4** **(*n =* 29)**	**Cluster 5** **(*n =* 31)**	***p*-value^**C**^**
28-day mortality—*N* (%)	5 (11.6%)	7 (20%)	4 (10%)	6 (20.7%)	7 (22.6%)	0.453 (0.049^*^)
Age years—median (Q1–Q3)^A^	59 (45–69)	59 (40–71)	57 (41–71)	60 (42–67)	61 (51–81)	0.463
Male gender—*N* (%)	34 (79%)	24 (67%)	23 (58%)	18 (62%)	22 (71%)	0.273
BMI—median (Q1–Q3)	27.4 (23.4–30)	23 (19.1–28.4)	24 (21.7–28.4)	25.3 (22.3–28.6)	25.7 (23.8–27.7)	0.118
ICU admission—*N* (%)	10 (23%)	13 (37%)	9 (23%)	5 (17%)	9 (29%)	0.412
Adequate antibiotic therapy—*N* (%) ^B^	41 (95%)	27 (77%)	37 (93%)	28 (97%)	30 (97%)	0.034
Biofilm-formation—median (Q1–Q3)	0.69 (0.56–0.8)	0.55 (0.44–0.8)	0.93 (0.7–1.15)	0.44 (0.34–0.52)	0.69 (0.57–0.94)	<0.0001
**Genetic markers—N (%)**						
MRSA	2 (5%)	8 (23%)	2 (5%)	0 (0%)	1 (3%)	0.005
*fnbA*	41 (95%)	34 (97%)	38 (95%)	28 (97%)	7 (23%)	<0.0001
*fnbB*	0 (0%)	1 (3%)	39 (98%)	0 (0%)	0 (0%)	<0.0001
*clfA*	42 (98%)	34 (97%)	40 (100%)	29 (100%)	29 (94%)	0.429
*clfB*	43 (100%)	35 (100%)	40 (100%)	29 (100%)	26 (84%)	0.0002
*fib*	43 (100%)	1 (3%)	38 (95%)	0 (0%)	2 (6%)	<0.0001
*vWbp*	43 (100%)	34 (97%)	39 (98%)	29 (100%)	25 (81%)	0.0010
**Coagulase gene size—*****N*** **(%)**						
547 bp	4 (9%)	2 (6%)	6 (15%)	27 (97%)	3 (10%)	
603 bp	12 (28%)	2 (6%)	31 (78%)	2 (3%)	3 (10%)	
660 bp	17 (40%)	30 (86%)	1 (3%)	0 (0%)	24 (77%)	0.0005
750 bp	8 (19%)	1 (3%)	0 (0%)	0 (0%)	1 (3%)	
875 bp	2 (5%)	0 (0%)	1 (3%)	0 (0%)	0 (0%)	
**Laboratory parameters—median (Q1–Q3)**						
CRP (mg/dL)	12.4 (8.3–24.3)	11.9 (6.8–21.9)	8.4 (3.1–24.9)	12.1 (4.7–26.4)	12.8 (6.8–19.4)	0.576
Leukocytes (G/L)	9 (6.1–13)	11.6 (8–16)	9.7 (4.9–13.9)	9.8 (5.8–13.7)	10.2 (7.8–12.9)	0.777
Platelets (G/L)	156 (108–241)	161 (100–331)	160 (105–245)	192 (129–272)	153 (105–217)	0.662
Hemoglobin (g/dL)	10.4 (9.2–11.7)	10.4 (9–12.2)	10.5 (8.7–12.2)	10.3 (9.4–12.2)	10.6 (9.2–12.3)	0.873
Creatinine (mg/dL)	1.2 (0.9–1.9)	1.2 (0.7–2)	1.2 (0.8–2.4)	1.03 (0.9–1.7)	1 (0.8–1.3)	0.484
Blood urea nitrogen (mg/dL)	25.6 (15.5–34.4)	21.3 (14.8–40.9)	25.4 (10.8–41.9)	20 (14.3–27.6)	19.8 (16.2–27)	0.828
Fibrinogen (mg/dL)	500 (360–669)	491 (410–618)	446 (335–625)	530 (302–679)	496 (370–600)	0.952
Prothrombin time (%)	68 (55–78)	73 (43–93)	76 (46–96)	68 (49–86)	75 (45–94)	0.993
Albumin (g/L)	29 (26.1–35.6)	30 (23.4–37.5)	31.6 (27.1–37.2)	31.5 (27.2–38.6)	29.3 (27.2–36.6)	0.618
Total bilirubin (mg/dL)	0.7 (0.5–1.3)	0.7 (0.4–3.3)	0.7 (0.5–1.2)	0.6 (0.3–1)	1 (0.6–1.6)	0.254

BMI, Body Mass Index, ICU: Intensive care unit; MRSA, Methicillin-resistant Staphylococcus aureus; COPD, chronic obstructive pulmonary disease; coa, coagulase; vWbp, von Willebrand factor-binding protein; clfA and clfB, clumping factor A and B; fnbA and fnbB, fibronectin-binding protein A and B; fib, fibrinogen-binding protein; ECDC, European Center of Disease Control; CRP, C-reactive protein.

* p-value for comparison of low (clusters 1 and 3) and high (clusters 2, 4, 5) mortality clusters were calculated using one-sided Fisher's exact test.

A Q1–Q3: Quartile 1 to Quartile 3.

B Adequate antimircobial treatment: defined as the initiation of at least one antimicrobial substance to which the isolated strain showed in vitro susceptibility according to the clinical breakpoints of the European Committee on Antimicrobial Susceptibility Testing (The European Committee on Antimicrobial Susceptibility Testing. Breakpoint tables for interpretation of MICs and zone diameters. Version 9.0, 2019) (EUCAST).

C * p-values were calculated by use of Kruskal-Wallis test for numeric values and Fisher's exact test for ordinal data*.

Between these five clusters no significant differences for laboratory parameters such as CRP (*p* = 0.82), white blood cell count (*p* = 0.96), platelet count (*p* = 0.31), hemoglobin (*p* = 0.75), creatinine (*p* = 0.29), blood urea nitrogen (*p* = 0.7), fibrinogen (*p* = 0.92), prothrombin time (*p* = 0.99), albumin (*p* = 0.76), total bilirubin (*p* = 0.07) or patients characteristics, including age (*p* = 0.44), BMI (*p* = 0.27), and ICU admission rates (*p* = 0.41) were observed. Mortality was significantly increased in the high risk group (cluster 2, 4, 5) compared to the low risk group (cluster 1, 3, *p* = 0.0497).

## Discussion

In this retrospective monocentric analysis of patients with SAB clinical and microbial data were gathered to investigate potential host-pathogen interactions by the use of a clinical Cox model and cluster analysis. Surprisingly, the clinical Cox model identified decreased fibrinogen levels as an independent risk factor for the 28-day mortality rate in our SAB patient collective.

One study performed by Schwameis et al. investigated the influence of fibrinogen in patients with bacteremia caused by various pathogens. That study showed no significant impact of fibrinogen on 30-day mortality, which might at least partially be explained by the low rates of *S. aureus* bacteremia of only 23% (Schwameis et al., 2015). Considering the major impact of *S. aureus* on human coagulation, mediated by the vast amount of virulence factors interacting with fibrinogen, the influence of fibrinogen in that study might have been underestimated. Other studies investigating large collectives of patients with SAB did not include fibrinogen in their analysis or studies investigating sepsis in general did show a link between fibrinogen and mortality but did not state any causative pathogen (Fowler et al., 2005; Cagatay et al., 2007; Forstner et al., 2013; Maeda et al., 2016; Holmes et al., 2018; Guilamet et al., 2019; Xia et al., 2020).

To unveil the underlying mechanism between fibrinogen and mortality in SAB patients, we implemented bacterial genetic markers, biofilm formation capacity and patients' characteristics in our clinical Cox model. However, none of these additional factors demonstrated a significant impact on 28-day mortality when calculating a pathogen adapted Cox model.

However, other prominent virulence factors like toxic shock syndrome toxin or staphylococcal enterotoxins, which were previously reported as independent risk factors for 30-day mortality, were not determined in the present study (Maeda et al., 2016).

By using cluster analysis, which was previously used to study the effect of genetic and biofilm formation capacity characteristics on patients‘ outcomes, the present study investigated the sole impact of bacterial genetic factors on mortality in a cohort of SAB patients (Cremers et al., 2019; Seymour et al., 2019).

We distinguished five different clusters by means of a PAM algorithm with different 28-day mortality rates, while patients' characteristics and laboratory parameters did not significantly vary between clusters. Clusters solely defined by genetic factors mainly varied in their coagulase gene sizes, *fnbB*, MRSA, and *fib* rates. The high mortality (>20%) in cluster 2 could be explained by the high MRSA content (23%), a high number of inadequate initial antimicrobial therapy (23%), and ICU admissions (37%) (Hanberger et al., 2011). Clusters 4 and 5, displaying a mortality rate >20%, only varied by means of coagulase gene size and *fib* rates.

Coagulase, one of the main aggregation and coagulation factors of *S. aureus*, interacts with fibrinogen and contributes to the formation of biofilms, fibrin shields, and microthrombi, subsequently explaining early intravascular infections and evasion of the innate immune system (Vanassche et al., 2012; Zapotoczna et al., 2015; Ko and Flick, 2016). Even though coagulase contributes to various aspects of bacterial virulence in experimental infection models, the lack of clinical data regarding fibrinogen as a surrogate parameter of mortality in SAB is surprising. Of interest, by further dividing clusters into low (≤ 400 mg/dL) and high fibrinogen (>400 mg/dL) levels, an even greater increase of mortality in clusters 2, 4, and 5 could be observed.

These low fibrinogen levels may reflect a tendency toward a coagulation activating state and therefore may explain observed higher mortality rates.

Another coagulation promoting factor in this complex interplay might be the accumulation of distinct coagulase gene sizes in specific high mortality clusters displaying a particular fibrinogen-binding motif within the tandem-repeat region of the coagulase gene. This region was previously shown to influence the enzyme's efficiency, which, together with the data obtained in the present study, possibly indicates a higher turnover of fibrinogen to fibrin by coagulases with gene sizes of 547 and 660 bp (Ko et al., 2016). However, on basis of this preliminary data a definite statement regarding the alteration of staphylococcal coagulase efficiency cannot be made, which outlines one of the limitations of the present study.

Intriguingly, clusters with low mortality (1 and 2) showed a high *fib* proportion compared to nearly no *fib* positive isolate in clusters with high mortality (2, 4, and 5). The overall prevalence of *fib* in our collective was 47%, which was lower when compared to other studies showing a range of 58–77% (Pérez-Montarelo et al., 2018). *Fib* is essential for biofilm induction and therefore increasing adhesion to vessels and other surfaces. Thus, while toxins are down-regulated, the expression of *fib* and other adhesins increases during the course of infection, enabling chronification and persistence while avoiding immunological defense mechanisms (Cardile et al., 2014; Tuchscherr and Löffler, 2016). Nevertheless, no publication showed a benefit of *fib* expression on survival up to now. Notably it must be emphasized that due to the limitations of the present study only an association between the genetic variations and mortality could be drawn, rather than a causal link.

Due to the retrospective study design and the patient population, which consisted of ICU patients and patients from the general ward, specific parameters like death due to infection, previous antibiotic treatment, parameters for calculation of the Sequential Organ Failure Assessment (SOFA) Score or laboratory parameters like D-dimer and thrombin-antithrombin complex could not be gathered. Nevertheless, our study population was comparable to other populations in SAB studies regarding e.g., malignancy and MRSA rates, age, creatinine, CRP, albumin and hemoglobin (Fowler et al., 2005; Cagatay et al., 2007; Hanberger et al., 2011; Forstner et al., 2013; Schwameis et al., 2015; Maeda et al., 2016; European Centre for Disease Prevention Control, 2018; Holmes et al., 2018; Guilamet et al., 2019).

In this retrospective cohort study, we found that coagulase gene size and presence of the *fib* gene appeared to be associated with a modified 28-day mortality of patients with *S. aureus* bloodstream infections. Prospective studies investigating bacterial markers of host-pathogen interactions, as well as studies investigating the molecular basis of these interactions are needed to confirm and explain the molecular basis of these findings.

## Data Availability Statement

The datasets generated for this study are available on request to the corresponding author.

## Ethics Statement

The studies involving human participants were reviewed and approved by the local ethics committee of the Medical University of Vienna, Austria (EK No. 1316/2017). Written informed consent for participation was not required for this study in accordance with the national legislation and the institutional requirements.

## Author Contributions

MKa and MKu contributed to clinical data acquisition and wrote the manuscript. MKa, MO, RK, VR, and HW contributed to laboratory experiments. FR has contributed to the statistical analysis. BJ, HL, and HB contributed to the study design. All authors have been contributing to revision of the manuscript.

## Conflict of Interest

FR was employed by Medical Diagnostics Laboratories. The remaining authors declare that the research was conducted in the absence of any commercial or financial relationships that could be construed as a potential conflict of interest.
