# Supplementary material for: Different Types of Coagulase Are Associated With 28-Day Mortality in Patients With Staphylococcus aureus Bloodstream Infections
Source: Front Cell Infect Microbiol. 2020 May 19;10:236. doi: 10.3389/fcimb.2020.00236 (PMC7248564; doi:10.3389/fcimb.2020.00236)
Supplement: Supplementary file 1 [file Data_Sheet_1.pdf]

Supplementary material:

Co-morbidities:

Malignancy was defined as active solid cancer, and leukemia was defined as an ongoing hematologic disease with or without ongoing chemotherapy.

Patients accounted for heart disease suffered from coronary artery disease, atrial fibrillation, heart insufficiency, cardiomyopathy, heart valve disease, or clinically relevant stenosis.

Renal impairment was defined as acute or chronic kidney failure, clinically relevant renal artery stenosis, acute tubulointestinal nephritis, or transplant failure.

Patients included into the liver cirrhosis group suffered from acute liver failure, chronic liver insufficiency, fatty liver disease or autoimmune hepatitis.

Autoimmune disease was classified as active rheumatoid diseases and autoimmune gastrointestinal diseases.

We defined anticoagulation therapy and platelet inhibiting therapy if patients received any medication inhibiting plasma anticoagulation or inhibiting thrombocyte function before the onset of bacteremia.

Table S1: Primers used

Primer Name	Sequence
**Coa1 (fwd)**	5‘–ATA GAG ATG CTG GTA CAG G–3‘
**Coa2 (rv)**	5‘–GCT TCC GAT TGT TCG ATG C-3‘
**clfA (fw)**	5’-ATT GGC GTG GCT TCA GTG CT-3’
**clfA (rv)**	5’-CGT TTC TTC CGT AGT TGC ATT TG-3’
**clfB (fw)**	5’-ACA TCA GTA ATA GTA GGG GCA AC-3’
**clfB (rv)**	5’-TTC GCA CTG TTT GTG TTT GCA C-3’
**fnbA (fw)**	5’-CAT AAA TTG GGA GCA GCA TCA-3’
**fnbA (rv)**	5’-ATC AGC AGC TGA ATT CCC ATT-3’
**fnbB (fw)**	5’-GTA ACA GCT AAT GGT CGA ATT GAT ACT-3’
**fnbB (rv)**	5‘-CAA GTT CGA TAG GAG TAC TAT GTT C-3‘
**fib (fw)**	5’-CTA CAA CTA CAA TTG CGT CAA CAG-3’
**fib (rv)**	5-GCT CTT GTA AGA CCA TTT TCT TCA C-3’
**vWBP (fw)**	5’-GCT GGA TTA AAT GGT GAA AGT CAT G-3’
**vWBP (rv)**	5’-GTT TAT TAA AAC GTT TTT GAT GAC C-3’

Genetic analysis

Coa PCR

PCR conditions for coa-PCR were as follows: One reaction contained 38 µl ddH$_2$O, 5 µl 10x buffer (Applied Biological Materials Inc., Canada), 1 µl dNTPs (10mM each) (Applied Biological Materials Inc., Canada), 0.1 µl of 100 pM forward primer (coa1), 0.1 µl of 100 pM reverse primer (coa2), 0.5 µl of 5 U/µl Taq-polymerase (5 U/µl) (Applied Biological Materials Inc., Canada) and 5 µl DNA. The following thermocycler (Thermocycler Peqstar 96U, peqlab, Germany) conditions were used: 95°C for 1 min, 30x (95°C for 30 sec, 57°C for 30 sec, 72°C for 53 sec), 72°C for 10 min and 8°C till further sample preparation.

The standardized *Staphylococcus aureus* strain ATCC33592 served as a positive control, displaying a *coa* gene size of 660bp

Virulence factor multiplex PCR

PCR conditions for the clotting factor multiplex PCR were as follows: One reaction contained 19.75 µl ddH$_2$O, 2.5 µl 10x buffer (Applied Biological Materials Inc., Canada), 0.5 µl dNTPs (10mM each) (Applied Biological Materials Inc., Canada), 0.05 µl of 100 pM of each forward primers (clfA-

fw, clfB-fw, fnbA-fw, fnbB-fw, fib-fw), 0.05 µl of 100 pM of each reverse primer (clfA-rv, clfB-rv, fnbA-rv, fnbB-rv, fib-rv), 0.25 µl of 5 U/µl Taq-polymerase (5 U/µl) (Applied Biological Materials Inc., Canada) and 1 µl DNA. The following thermocycler (Thermocycler Peqstar 96U, peqlab, Germany) conditions were used: 92°C for 1 min, 35x (91°C for 1 min, 51°C for 1 min, 72°C for 1 min), 72°C for 3 min and 8°C till further sample preparation.

<u>vWbp PCR</u>

PCR conditions for vWbp-PCR were as follows: One reaction contained 20.65 µl ddH_2O, 2.5 µl 10x buffer (Applied Biological Materials Inc., Canada), 0.5 µl dNTPs (10mM each) (Applied Biological Materials Inc., Canada), 0.05 µl of 100 pM forward primer (vWBP-fw), 0.05 µl of 100 pM reverse primer (vWBP-rv), 0.25 µl of 5 U/µl Taq-polymerase (5 U/µl) (Applied Biological Materials Inc., Canada) and 1 µl DNA. The following thermocycler (Thermocycler Peqstar 96U, peqlab, Germany) conditions were used: 92°C for 1 min, 35x (92°C for 1 min, 48°C for 1 min, 72°C for 1 min), 72°C for 3 min and 8°C till further sample preparation.

<u>RFLP</u>

Restriction fragment length polymorphism was assessed using the following reaction conditions: One reaction contained 12.3 µl H_2O, 2.5 µl buffer A (New England Biolabs Inc., Germany), 0.2 µl of 10 U/µl AluI (New England Biolabs Inc., Germany) and 10 µl PCR product. The reaction was performed at 37°C for 1 h 30 min.
