# Supplementary material for: Different Types of Coagulase Are Associated With 28-Day Mortality in Patients With Staphylococcus aureus Bloodstream Infections
Source: Front Cell Infect Microbiol. 2020 May 19;10:236. doi: 10.3389/fcimb.2020.00236 (PMC7248564; doi:10.3389/fcimb.2020.00236)
Supplement: Supplementary file 2 [file Image_1.pdf]

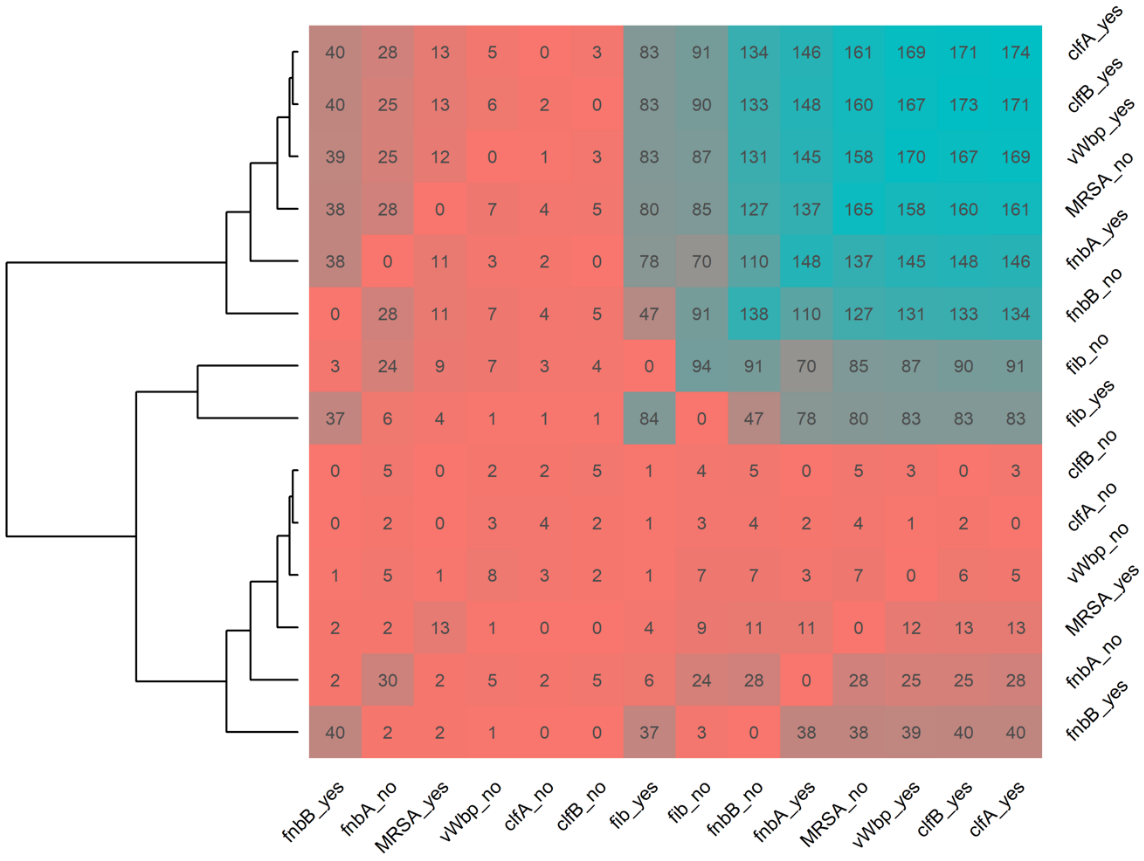

Figure S1: Genetic trait heat-map of invasive *Staphylococcus aureus (S. aureus)* isolates analyzed within this study. MRSA, Methicillin-resistant *Staphylococcus aureus*; *coa*, coagulase; *vWbp*, von Willebrand factor-binding protein; *clfA* and *clfB*, clumping factor A and B; *fnbA* and *fnbB*, fibronectin-binding protein A and B; *fib*, fibrinogen-binding protein
