# Supplementary material for: Different Types of Coagulase Are Associated With 28-Day Mortality in Patients With Staphylococcus aureus Bloodstream Infections
Source: Front Cell Infect Microbiol. 2020 May 19;10:236. doi: 10.3389/fcimb.2020.00236 (PMC7248564; doi:10.3389/fcimb.2020.00236)
Supplement: Supplementary file 3 [file Image_2.pdf]

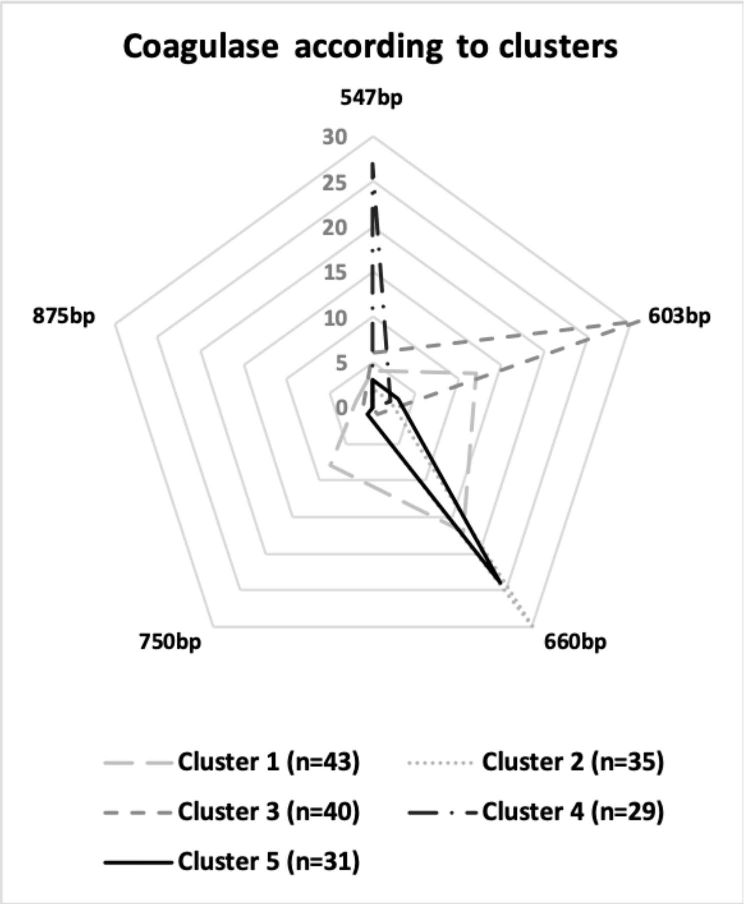

Figure S2: Radar Chart of different coagulase gene sizes within different clusters of patients with *Staphylococcus aureus* bacteremia. High mortality clusters 2, 4, and 5 showed a high proportion of isolates with coagulase gene sizes 547basepairs (bp) and 660-bp. The low mortality cluster 1 displayed a more disseminated genetic setup, while cluster 3 included more isolates with a coagulase gene size of 603bp.
